# Effects of Pangasius (*Pangasius hypophthalmus*) and Skipjack Tuna (*Sarda orientalis*) mince blend on the quality of fish products: Ways to utilize resources and nutrition in Bangladesh

**DOI:** 10.1002/fsn3.2612

**Published:** 2021-09-29

**Authors:** Md. Sazedul Hoque, Shatabdi Roy, Shihab Sharar Mukit, Md. Bokthier Rahman, Shaida Akter

**Affiliations:** ^1^ Department of Fisheries Technology Faculty of Fisheries Patuakhali Science and Technology University Patuakhali Bangladesh; ^2^ WorldFish Center ECOFISH‐II Project Dhaka Bangladesh; ^3^ Department of Fisheries Management Bangabandhu Sheikh Mujibur Rahman Agricultural University Salna Bangladesh; ^4^ Bangladesh Fisheries Research Institute Freshwater Station Saidpur Bangladesh

**Keywords:** fish mince blend, nutritional security, resource utilization, textural quality, value‐added fish products

## Abstract

This study was conducted with the aims of utilizing the resources and analyzing the quality of value‐added fish products, fish ball made from a fish mince blend of pangasius (*Pangasius hypophthalmus*) and skipjack tuna (*Sarda orientalis*) (ratios P_100_:T_0_, P_75_:T_25_, P_50_:T_50_, P_25_:T_75_, P_0_:T_100_), under two cooking processes (two‐step heating and autoclaving). The textural quality (softness/firmness [S/F]; chewiness/rubberiness [C/R]; and folding test [FT]) and the nutritional quality of the fish products were determined by the sensory method and AOAC method, respectively. The results showed that tuna had higher utilization than pangasius. The products from the washed fish mince blend showed better textural properties with two‐step heating (50℃ for 60 min and 100℃ for 30 min) than with autoclaving (120℃, 15Ib/Inc^2^ for 15 min) and unwashed process. Of the five fish mince blend ratios, P_50_:T_50_ showed significant higher textural properties (FT, S/F, and C/R values) than the other ratios (*p* < .05). Further improvements in textural qualities were observed when the mince blend was washed with different salt solutions (0.1% NaCl, KCl, CaCl_2_, and MgCl_2_). Whitish or bright color attributes were obtained from pangasius mince, which became darker proportionately with increasing proportions of tuna mince (*p* < .05). The texture, color, and nutritional quality of the fish products were affected by washing, heating, and the compositional differences in the fish species. Thus, value‐added fish products based on a fish mince blend could contribute to an increase in total resource utilization and nutritional security in Bangladesh.

## Graphical abstract

Tuna meat mince had a higher yield than pangasius meat mince. Pangasius and tuna at P_50_:T_50_ ratio showed better textural quality than other ratios. The heating process, salt solutions washing and muscle compositions significantly affects the color and textural quality of the fish ball.

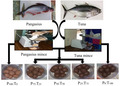

**Key Findings**

- Tuna meat mince had higher yield than pangasius meat mince.
- Pangasius and tuna at P_50_:T_50_ ratio showed better textural quality than other ratios.
- Heating process, salt solutions washing, and muscle composition significantly affect the color and textural quality of fish ball.

## 1 INTRODUCTION

In Bangladesh, fish and fisheries contributes to food and nutritional security by accounting for 3.61% of the Gross Domestic Product (GDP), 24.41% of Agricultural GDP, 1.51% of total export earnings, 60% of the supply of animal protein, and 11% of employment (Department of Fisheries [DoF], 2018). As a result of consumers’ increasing awareness of health and the demand for high nutrition, consumption of fish and fish products is increasing daily. Currently, value‐added mince/surimi–based fish products could meet the existing nutritional demands of consumers and contribute to the benefits of fish‐processing industries of Bangladesh (Nowsad et al., 2000). Several studies report that fish ball is an important mince/surimi‐based value‐added fish product (Affandi et al., 2019; Alkuraieef et al., 2020; Chowdhury et al., 2017; Ibrahim, 2015; Asikin et al., 2020; Zhao et al., 2019). Among the foods that are “ready to eat” or “ready to cook,” fish ball is a popular and tasty value‐added fish product (Dutta, 2009). In recent years, consumers’ preferences have moved significantly towards foods that are ready to eat because of rapid urbanization, an increase in the population of women working (Akter et al., 2013), and increased purchasing capacity of consumers (Hoque & Begum, 2016). Working people along with the new generation of students and young people are now more interested in ready‐to‐eat foods to save time and energy (Akter et al., 2013). Unfortunately, fish meat–based value‐added fish products are not commercially produced in Bangladesh. With the exception of some initiatives, producing value‐added fish products at a commercial level in Bangladesh is not possible due to a lack of entrepreneurship, the unavailability of raw materials, and the variability in consumer behavior (Ejaz et al., 2009).

Recently, there have been significant quantities of low‐cost fresh water fish (LFWF) and low‐cost marine water fish (LMWF), and this has received adequate attention for value addition. Individual catches of pangusius (*Pangasius hypophthalmus*) and meso‐pelagic/pelagic marine skipjack tuna (*Sarda orientalis*) are readily available and priced cheaply in the market, and this could serve as an adequate source of raw material for preparing value‐added fish products such as fish ball. In Bangladesh, pangasius production is absorbed by the domestic markets, and because it has become one of the cheapest fish species available in the domestic markets, it is eaten mainly by consumers in lower income brackets (Anwar, 2011). Pangasius in Bangladesh may provide an undesired muddy flavor (Mikael et al., 2014) which could be minimized by using different spices and local ingredients in preparing it for value‐added products. In addition, when compared with freshwater fish, the consumer preference for marine fish tuna was lower, as was its price at the market (Belton et al., 2011). As a consequence, large marine resources in Bangladesh were used to produce huge tuna which were mostly not welcomed by domestic consumers, so it is not being preferred as food. Value‐added fishery products enable the processing of fish species that are less desirable for consumers but could contribute greatly to consumer nutrition and economics (Çaglak, 2018). This suggests that further value‐addition is required for tuna and pangasius. In addition, due to the availability of raw materials, surimi industries based on single fish such as sardine, mackerel, tuna, and Alaska Pollock have been developed in many countries. However, there is no surimi/mince‐based commercial industry (single or multiple fish species) in Bangladesh. Fish ball (Hoque et al., 2007) and fish sausage (Nowsad & Hoque, 2009), made from mixed‐mince of five low‐cost marine fish, have been reported in experiments. Effectively utilizing resources by producing diversified surimi/mince‐based value‐added fish products from underutilized/low‐priced fish species will bring immediate benefits to the existing use of resources, to fish processing industries, and to consumers in Bangladesh (Nowsad, Hoque, et al., 2000). Therefore, the mixed catches of pangasius and tuna could provide a substantial volume of mince and provide acceptable and available source of nutrition, taste, and flavor.

There are a number of other factors that influence the gel‐forming ability of fish surimi/mince‐based products. High fat content in the fish muscle weakens the ability to form gel (Suzuki & Watabe, 1987). Washing is necessary to remove water‐soluble substances, mainly sarcoplasmic proteins, fat, and other undesirable materials such as pigments. Washing can be done with different types of salt solution. The function of salt in the solution is to help solubilize myofibrillar proteins to improve gel formation (Niwa, 1992; Okada, 1986). When the protein unfolds, sodium cation interacts with the anionic groups on several amino acids. The cation‐anion interaction between carboxyl groups on the amino acids of fish proteins and the sodium ions may inhibit unfolding of the protein and the exposure of bonding sites important for the gelation process (Chung et al., 1993). Upon heat treatment (boiling, broiling, or deep‐frying), the fish products (for example, fish ball) from mixed surimi/mince may develop an elastic texture and a nice feeling in the mouth that can conceal the fishy or muddy odor in the produced products (Nowsad et al., 2000). There have been several studies conducted to develop value‐added fish products such as fish ball from both single freshwater species: tilapia (*Oreochromis mossambicus*) (Mugale et al., 2015), rohu (*Labeo rohita*) (Dutta, 2009), common carp (*Cyprinus carpio*) (Abdel‐Aal et al., 2014), striped catfish (*Pangasianodon hypophthalmus*) (Akter et al., 2013), and threadfin bream (*Nemipterus tolu*) (Yu, 1994); and marine water species: Indian mackerel (*Rastrelliger kanagurta*) (Alkuraieef et al., 2020), Eastern little tuna (*Euthynnus affinis*) (Affandi et al., 2019), mosul bleak (*Alburnus mossulensis*) (Duman & Peksezer, 2016), Spanish mackerel (*Scomberomoru guttatus*) (Tee & Siow, 2017), and sea catfish (*Tachysurus thalassinu*s) (Nowsad, Hoque, et al., 2000). Hoque et al. (2007) prepared fish ball from a mince mixed with marine red jewfish (*Johnius argentatus*), sea cat fish (*Tachysurus thalassinus*), jeweled shad (*Ilisha filigera*), horse mackerel (*Megalaspis cordyla*), and skipjack tuna (*Sarda orientalis*). Numerous studies reported that nutritional, textural, and sensory characteristics of fish balls improve with different additives: carrageenan (Asikin et al., 2020), fish protein isolates (Ibrahim, 2015), rice bran flour (Affandi et al., 2019), tapioca and potato starch (Tee & Siow, 2017), potato flour (Chowdhury et al., 2017), seaweed (Loso & Pascual, 2020), and Perilla (*Perilla frutescens*) leaf extracts (Zhao et al., 2019). However, there is no study on surimi/mince‐based value‐added products such as fish ball made with a mixture of meat from fresh water and marine water fish species. Therefore, preparation of value‐added fish products from a mixed mince of fresh water and marine water fish could be promising for utilizing resources and consumers’ nutritional security. The experiments in this study aim to examine the effects of different ratios of pangusius and tuna in a mince blend, and to evaluate the quality characteristics (sensory and biochemical) of value‐added fish products that have been affected by different washing and heating treatments.

## 2 MATERIALS AND METHODS

### 2.1 Fish species

The meat from low‐cost white muscle freshwater pangusius (*Pangasius hypophthalmus*) and low‐cost dark muscle marine water skipjack tuna (*Sarda orientalis*) was used to produce a mince blend and the resulted product, fish ball. Both fish species were collected from a local market at Patuakahli and transferred to the laboratory on ice and stored frozen (−18℃) until use.

### 2.2 Preparation of fish mince blend

The frozen fish were thawed under running tap water. The fish were weighed and then washed with clean water, before being skinned and filleted. Then mince was produced with a mechanical mincer (Panasonic MK‐MG 1,300, Malaysia) through an orifice of 1 millimeter (mm) in diameter so that all the bones and connective tissues were removed from the muscles of the fish. All the utensils used in the experiment were cleaned with distilled water. The mince was then divided into two equal portions. One portion was kept unwashed and the other was washed with distilled water. From both portions, five mince blends were prepared at ratios of 100:0, 75:25, 50:50, 25:75, and 0:100, respectively. The entire process from mincing the raw fish to producing the fish ball was done at about 5℃ to 8℃. The low temperature was maintained with sufficient ice around the fish, flesh, and mince.

### 2.3 Preparation of spices

Various ingredients were incorporated into the mixed mince to prepare the fish ball. For this purpose, the following spices, purchased from the local market, were used: onion, garlic, ginger, cinnamon, clove, red pepper, and black pepper. The spices were dried in an air oven (DO‐35, Human Lab Instrument Co, Korea) at 50℃ for 24 h. The dried spices were ground with a mechanical grinder (Miyako, DL‐718, China) to make a powder which was then sieved by a fine mesh metallic sieve (35 mesh, 500um, Fieldmaster^®^). The resulting powdered spices were poured into small labeled plastic pots and stored in a refrigerator at 4℃. The level of various ingredients and species used for fish ball preparation is given in Table 1 and Table 2.

**TABLE 1:** Percentage of ingredients used to prepare the fish balls

Ingredients	%
Table salt (NaCl)	2–2.5
Sugar	1.60
All spices (Table 2)	1.50
MSG	0.10
Starch	10.0
Color (red and orange asthaxanthin)	0.04
Vegetable oil	2.00
Water	10.0

**TABLE 2:** Percentage of spices used to prepare the fish balls

Spice	% (of a spice total of 1.5%)
Red pepper powder	30
Onion	20
Garlic	20
Ginger	10
Clove	5.0
Cinnamon	5.0
Black pepper	10

### 2.4 Preparation of fish balls

Each fish ball was prepared following the method of Nowsad, Hoque, et al. (2000) and Hoque et al. (2007) in which the mince blends were combined with 2.0% NaCl, 1.6% sugar, 1.0% spices (ginger, garlic, onion, and chili powder), 0.1% MSG, 10% starch soluble, and 2% vegetable oil. Mixing was done for a total of 16 min. First, the mince were pounded with salt for 5 min. Following this, the sugar, spices, and starches were added to the mince and ground for 4 min. Finally, vegetable oil was incorporated and the mince was grounded again for 7 min. The resulting ground paste was shaped into balls maintaining pangasius‐skipjack tuna ratios of 100:0, 75:25, 50:50, 25:75, and 0:100. The fish balls were kept at room temperature for 1–2 h before the quality analysis. The quality of each fish ball was determined under two different cooking processes: two‐step heating (incubation at 50℃ for 60 min and then heating at 100℃ for 30 min) and autoclave (BIOBASE, BKQ‐B50II, Biobase Biodustry Co., Ltd, Shandong, China) cooking (120℃ at 15 lbs/inch^2^ for 20 min).

### 2.5 Effects of washing with different salt solutions on the quality of fish balls

A separate study was conducted to measure the effects of washing on the fish ball. The mince was washed using different salt solutions (0.1% NaCl, KCl, CaCl_2,_ and MgCl_2_) as suggested by Hossain et al. (2004). After washing the mince with the different salt solutions, comparisons were made with mince washed with distilled water and mince unwashed. Necessary ingredients were added and each fish ball was prepared following the methods described in the previous section.

### 2.6 Sensory analysis of fish balls

A panel of nine, comprising graduate students and academic staff of the Department of Fisheries Technology of Patuakhali Science and Technology University, provided sensory assessments of the products (Nowsad, Kanoh, et al., 2000). Prior to testing, the panelists were familiarized with the properties of meat gel and were given instructions for scoring the samples. Pretests were undertaken with selected samples to familiarize the panelists with the measurement procedure. Three discs of gel (0.5 cm thick) were given to each panelist, so they could recognize every attribute. Softness/firmness (S/F) was defined as the amount of force required to bite through the sample with incisors, and chewiness/rubberiness (C/R) was defined as the amount of effort a panelist had to exert in chewing to prepare the sample for swallowing.

The sensory quality was evaluated by numerical scores from 1 to 10. For S/F, 1 = very soft and 10 = extremely firm; for C/R, 1 = not chewy/rubbery and 10 = extremely chewy/rubbery. A folding test was carried out by folding a 2 mm thick sample disc into halves and quarters as per the method developed by Nowsad, Kanoh, et al. (2000). The scale used was A^++^ = no crack when folded into quarters, A^+^ = no crack when folded into half but a crack when folded into quarters, A = a crack when folded into half, and B^+^ = broken and split into halves when folded.

### 2.7 Biochemical test

The triplicate samples of unwashed mince, washed mince, mince blend, and cooked fish balls were analyzed for proximate composition such as crude protein, crude lipid, moisture, and ash content. The composition was analyzed according to the standard procedure of the Association of Official Analytical Chemists (AOAC, 2000).

### 2.8 Statistical analysis

The data obtained were subjected to analysis of variance (ANOVA) and the mean comparisons were carried out by Duncan's Multiple Range Test using SPSS package software (SPSS 16.00 for windows, SPSS Inc.). A significant difference was defined at *p* < .05.

## 3 RESULTS AND DISCUSSION

### 3.1 Total utilization of wet fish

The utilization of wet fish, fish mince, by‐products, and weight loss due to washing is presented in Table 3. The study showed that meat utilization was higher in tuna than pangasius. The amount of by‐products produced was 55.45% and 45.38% for pangasius and tuna, respectively. Tuna produced 10% more yield of mince production than pangasius. The total mince was separated into two groups at 50:50 ratios to prepare fish ball from unwashed (UW) and washed mince (WM). The washing process showed significant weight loss, irrespective of the species. However, the washing process resulted in greater weight loss for pangasius mince (4.08%) than for tuna mince (3.66%) (Table 3). The individual characteristics of the fish muscles were a factor in weight loss: higher fat content in the white muscles of pangasius meat resulted in greater weight loss. On the other hand, the dark muscles and lower fat content in tuna resulted in less weight loss after washing. After the washing and dewatering process, 40%–50% of solids could be lost from the minced fish (Bakli et al., 2020). The result suggested that a significant amount (45%–55%) of by‐products are produced from inedible parts of the fish, which could be further utilized for preparation of other products, such as fish meal and fish silage. Ibrahim (2015) found that the fish‐processing industry produces more than 60% by‐products as waste, including head, skin, trimmings, fins, frames, viscera, and roes; the remaining 40% is edible fish muscle for human consumption. Department of Fisheries (DoF, 2013) reported that 25%–30% of fish comprises scales, guts, fins, bones, heads, and shells, which are discarded as waste during processing or preprocessing. On the other hand, in the washing process, there is a loss of valuable proteins, lipids, and minerals which are beneficial for consumer health. Losing such valuable nutrients in the name of improving textural quality is thought to be a serious waste of resources (Nowsad & Hoque, 2009). The by‐products and waste production varies based on fish species and size, the season, the postharvest or industrial preparation processes, and solid wastes generated from seafood factories (Ibrahim, 2015). In this study, efforts were made to utilize both the washed and unwashed mince from pangasius and tuna. By using the unwashed mince, unwanted loss of valuable nutrients could be minimized. Doing so, it would also substantially reduce the cost of production by increasing the weight of the final products, and the loss of materials would be low. However, the quality of unwashed minced‐based products and their shelf‐life in storage is a concern when compared with products from washed mince.

**TABLE 3:** Total utilization of wet fish and fish mince

Fish Species	Wet Fish				Fish Mince		
	Wt. of whole fish (g)	Wt. of mince (g)	Wt. of by‐product (g)	By‐products (%)	Unwashed Mince (g)	Mince after washing (g)	Weight loss (%)
Pangasius	2,200	980	1,220	55.45	490	470	4.08
Skipjack Tuna	1,300	710	590	45.38	355	342	3.66

### 3.2 Textural properties of fish balls

The textural qualities of the fish balls prepared from both unwashed and washed mince blends and cooked via two different cooking processes are shown in Table 4. For the fish ball from the unwashed mince blend, the lowest S/F and C/R score was observed in the mince with the ratio of P_100_:T_0_ for both two‐step heating and autoclaving. For the folding test, P_100_:T_0_ also showed the lowest score (*p* < .05) in two‐step heating. However, autoclaving showed the lowest folding test score for P_25_:T_75_. The ratio of P_0_:T_100_ had significant higher (*p* < .05) textural properties (S/R, C/F and Folding test) than P_100_:T_0_ in both heating processes, which was similar to P_50_:T_50_ (*p* > .05). In two‐step heating, the higher S/F, C/R and FT scores were observed for P_50_:T_50_ than any other ratio of fish mince. In autoclaving, the fish ball from the P_50_:T_50_ ratio showed higher S/R and folding test scores; however, higher C/R scores were found in P_75_:T_25_. In case of autoclaving, significant variations in textural properties were observed in fish balls from different ratios of fish mince. Of the different ratios of mixed mince, the unwashed mince at P_50_:T_50_ ratio put through the two‐step heating process showed better textural properties than any other ratio and heating treatment used. Affandi et al. (2019) investigated the texture properties (cohesiveness, chewiness, and springiness) of eastern little tuna fish ball (*Euthynnus affinis*) and found that they were affected by the substitution of rice bran and tapioca flour. The eastern little tuna fish ball with 25% rice bran and 75% tapioca flour had better textural properties and was more acceptable than other formulas. Hossain et al. (2004) reported better gel properties for both silver carp (*Hypophthalmichthys molitrix*) and pangasius (*Pangasius hypophthalmus*) under the two‐step heating process than one‐step heating.

**TABLE 4:** Textural characteristics of the fish balls under two different heating treatments

Heating Treatment	Mince Ratio	Unwashed Mince			Washed Mince		
		S/F	C/R	FT	S/F	C/R	FT
Two‐step heating	P_100_:T_0_	5.07 ± 0.12^d^	3.67 ± 0.58^c^	4.30 ± 0.20^d^	4.67 ± 0.15 ^b^	6.07 ± 0.12^b^	5.37 ± 0.12^c^
	P_75_:T_25_	5.80 ± 0.10^b^	6.83 ± 0.76^ab^	8.27 ± 0.31^b^	4.37 ± 0.21^b^	5.43 ± 0.31^c^	7.30 ± 0.20^b^
	P_50_:T_50_	6.10 ± 0.10^a^	7.63 ± 0.15^a^	9.33 ± 0.42^a^	6.40 ± 0.20^a^	8.47 ± 0.45^a^	9.10 ± 0.17^a^
	P_25_:T_75_	5.53 ± 0.15^c^	6.50 ± 0.50^b^	7.37 ± 0.15^c^	3.33 ± 0.15^b^	4.63 ± 0.47 cd	4.03 ± 0.15^d^
	P_0_:T_100_	5.93 ± 0.21^ab^	6.87 ± 0.15^ab^	7.43 ± 0.21^c^	4.63 ± 0.31^c^	5.00 ± 0.00^d^	4.27 ± 0.12^d^
Autoclaveheating	P_100_:T_0_	5.00 ± 0.00^d^	3.97 ± 0.95^c^	5.63 ± 0.15^b^	4.37 ± 0.15^c^	7.30 ± 0.15^a^	9.10 ± 0.15^a^
	P_75_:T_25_	5.57 ± 0.32^bc^	7.60 ± 0.10^a^	5.17 ± 0.12^c^	3.20 ± 0.15^d^	4.03 ± 0.15^d^	3.37 ± 0.15^c^
	P_50_:T_50_	5.70 ± 0.10^ab^	6.53 ± 0.15^b^	6.20 ± 0.17^a^	6.07 ± 0.15^a^	6.63 ± 0.15^b^	9.17 ± 0.15^a^
	P_25_:T_75_	5.33 ± 0.15^c^	6.57 ± 0.06^b^	3.50 ± 0.44^d^	3.93 ± 0.15^c^	3.60 ± 0.15^e^	3.30 ± 0.15^c^
	P_0_:T_100_	5.97 ± 0.15^a^	6.80 ± 0.10^b^	6.23 ± 0.21^a^	5.33 ± 0.15^b^	5.73 ± 0.15^c^	5.30 ± 0.15^b^

Note

Mean ± *SD* (*n* = 9); The letters in the columns indicate the significant differences (*p* < .05) of textural properties from different mince blend ratios under the same heating treatment. “P” and “T” represent the fish meat pangasius and tuna, respectively.

For the fish ball prepared from the washed mince blend, significant variations in textural properties were observed with both the two‐step and autoclaving heating processes. In both heating process, fish balls comprising a single fish mince or a higher proportion of mince from the same species had lower textural characteristics than the fish ball with a ratio of P_50_:T_50_ (*p* < .05). Significantly higher S/F, C/R, and FT scores were observed for the P_50_:T_50_ ratio when the two‐step heating process was used (*p* < .05) than with the autoclave pressure heating. This was supported by the study of Hossain et al. (2004). Sarika et al. (2020) also observed that high pressure (200MPa for 15 min) can retain the softer/elastic texture of fish balls while conventional heat (90^◦^C for 60 min) causes a harder and drier texture. The P_100_:T_0_ showed similar textural characteristics (FT scores) to P_50_:T_50_ when the autoclaving heating process was used (*p* > .05).

In general, the mixed mince ratio of P_50_:T_50_ with the two‐step heating process produced a better fish ball compared to those of other ratios and heating treatments. The results clearly show that the textural quality of fish balls from a mixed mince blend was influenced greatly by the dominant fish species, the ratio used in the mince blend, and the washing and heating treatments used. Hoque et al. (2007) found that the two‐step cooking process resulted in a very good textured fish ball. It was revealed that fish balls made from unwashed mince were firmer than fish balls made from washed mince. Additionally, Kongpun (1999) reported that washed mince had a better gel forming ability than unwashed mince. The textural differences might be due to the presence of individual fish mince in each ratio and their muscle properties. A similar study was conducted with five low‐cost marine fish (red jewfish, sea cat fish, jeweled shad, horse mackeral, and skipjack tuna). In the study, a mince blend was produced comprising 15%–30% of individual fish minces and it found that the mince blend with a higher proportion of red jewfish, sea cat fish, and horse mackerel showed the highest gel strength (Hoque et al., 2007). Alkuraieef et al. (2020) also found a better textural quality score in fish balls made from washed mince of Indian mackerel. Çaglak (2018) suggested white meat fish for better textural quality of surimi‐based products. The washing process eliminated sarcoplasmic proteins, enzymes, lipids, blood, minerals, and other organic and nitrogenous compounds from the mince, and this improved the textural quality by concentrating the myofibrillar protein (Lee, 1984; Roussel & Cheftel, 1988). Similar results have been reported by many authors working with other fish species (Akter et al., 2013; Babbitt et al., 1985; Webb et al., 1985).

### 3.3 Color properties of fish balls

**FIGURE 1:**
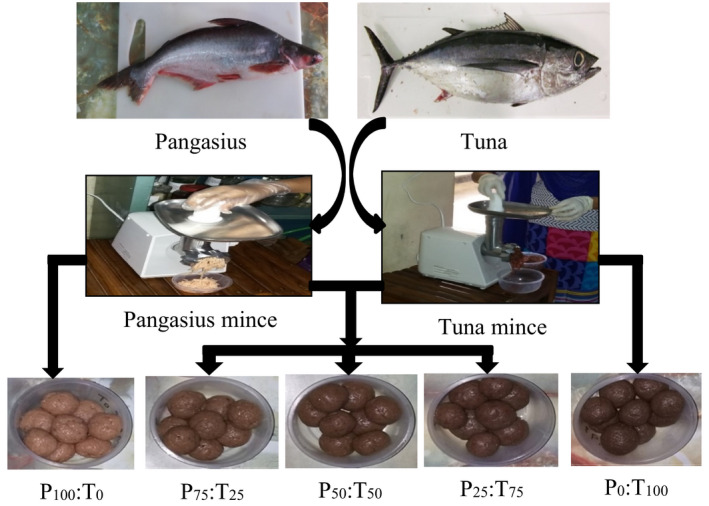
Preparation of the fish balls from different ratios of mixed unwashed mince blends. ['P', pangasius, 'T', tuna, the numbers represent the amount of fish mince (%) from each.]

The color properties of the fish balls prepared from both unwashed and washed mince blends and cooked under two different heating treatments are presented in Table 5. Of the different mince ratios used for the fish balls, the mince comprising solely of pangasius (P_100_:T_0_) showed the most brightness or whiteness in color. On the other hand, the mince comprising solely of tuna (P_0_:T_100_) had the darkest color, irrespective of the heating process used (Table 5 and Figure 1). As the amount of tuna in a ratio increased, a gradual increase in darkness and a simultaneous gradual significant decrease (*p* < .05) in whiteness were observed with both heating processes. The results indicate that mince comprising solely of pangasius or mince mixed with a greater proportion of pangasius had a brighter color than other ratios (Table 5 and Figure 1). Similar trends in changes in color were observed for both washed and unwashed mince. White meat and washing are the important quality (color) characteristics for surimi products (Çaglak, 2018). The white and dark muscle properties of pangasius and tuna, respectively, might be responsible for this result. For all mince blends, regardless of ratios, washed mince produced a brighter color than unwashed mince under both heating processes (*p* < .05). The washing process had the effect of brightening the color of the mince by removing sarcoplasmic protein, fat, other dissolved nutrients, and color components. This result is supported by the work of Yu (1994) who found that the color of fish balls made from twice and thrice washed minces was significantly whiter than for fish balls made from unwashed mince and mince washed only once. Huda et al. (2010) reported that the washing process increased the lightness of surimi‐based products such as commercial fish balls. Sensory evaluation of balls from washed Indian mackerel mince revealed good scores for color, odor, taste, and acceptability (Alkuraieef et al., 2020). In general, autoclave heating produced fish balls with slightly less brightness (a brownish color) than the two‐step heating process. This might have resulted from brown polymers (melanoidins) forming on the products in the high heating (autoclaved) process (Starowicz & Zieliński, 2019). The eastern little tuna fish ball developed a brownish color when substituted with 25% rice bran and 75% tapioca flour (Affandi et al., 2019), and the least amount of seaweed added produced a golden brown color (Loso & Pascual, 2020).

**TABLE 5:** Color of the fish balls under two different heating treatments

Heating Treatment	Mince Ratio	Color	
		Unwashed	Washed
Two‐step heating	P_100_:T_0_	9.00 ± 0.00^aB^	9.22 ± 0.12^aA^
	P_75_:T_25_	7.57 ± 0.49^bB^	9.05 ± 0.00^bA^
	P_50_:T_50_	6.77 ± 0.32^cB^	8.03 ± 0.06^cA^
	P_25_:T_75_	4.23 ± 0.15^dB^	4.80 ± 0.38^dA^
	P_0_:T_100_	3.25 ± 0.20^eB^	3.67 ± 0.15^eA^
Autoclaving	P_100_:T_0_	8.35 ± 0.35^aB^	9.07 ± 0.20^aA^
	P_75_:T_25_	7.62 ± 0.30^bB^	8.83 ± 0.25^aA^
	P_50_:T_50_	6.50 ± 0.50^cB^	7.37 ± 0.10^bA^
	P_25_:T_75_	4.03 ± 0.10^dB^	4.40 ± 0.25^cA^
	P_0_:T_100_	3.07 ± 0.12^eB^	3.60 ± 0.15^dA^

Note

Mean ± *SD* (*n* = 9); The lowercase letters in the columns indicate the significant differences (*p* < .05) in the color of the fish balls from different mince blend ratios under the same heating treatment. The uppercase letters in each row indicate the significant differences (*p* < .05) in the color of the fish balls from different mince blend ratios under different washing treatments.

For the sensory quality characteristics, the fish balls prepared with washed mince at a ratio of P_50_:T_50_ and cooked with the two‐step heating showed better textural and color properties. Therefore, P_50_:T_50_ washed mince under two‐step heating was selected for further study. The further study (section 3.4) investigated the effects on the sensory and biochemical characteristics of fish balls of washing with different salt solutions (0.1% of NaCl, KCl, CaCl_2_, and MgCl_2_) compared with using distilled water (DW) for washing and using unwashed mince.

### 3.4 Effects of washing on texture quality of fish ball

**FIGURE 2:**
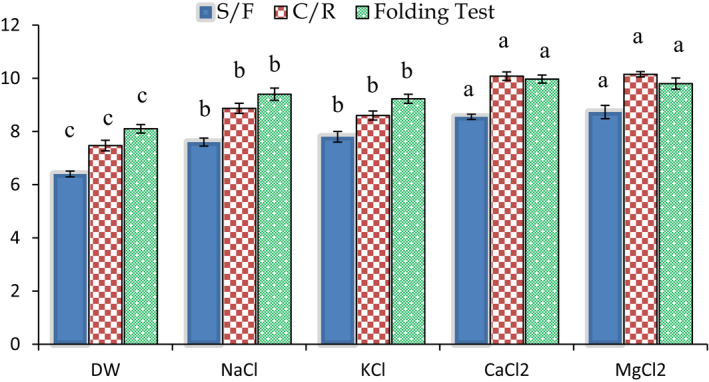
The effects of washing with different salt solutions on the textural characteristics of the fish balls

The textural characteristics of the fish balls from a P_50_:T_50_ ratio and washed with different salt solutions are presented in Figure 2. From the results, it was observed that the textural properties (S/F, C/R, and FT value) were better in fish balls prepared from the mince blend washed with 0.1% salt solution (NaCl, KCl, CaCl_2_, and MgCl_2_) than with DW (*p* < .05). The score for textural quality increased significantly for NaCl and KCl compared to DW (*p* < .05), and this was further increased for CaCl_2_ and MgCl_2_ (*p* < .05). However, similar textural properties were found in fish balls from mince washed with a NaCl and KCl solution (*p* > .05) and a CaCl_2_ and MgCl_2_ solution (*p* > .05). The results found that washing with different types of salt solutions (at appropriate concentrations) had a greater influence on the final fish products than washing with distilled water. This might be due to the differences in the chemical structure of salt (monovalent and divalent) and its interaction with the protein structures of the fish. The chemical structure of monovalent NaCl salt, with its ionic strength being higher than that of KCI, may form a salt bridge with the amino and carboxyl side of the protein which may have resulted in a better textural quality. The current result from this experiment is similar to the results from several other studies. Higher gel‐forming abilities were observed when fish mince fish was washed with 0.1% NaCl than with other concentrations (Hossain et al., 2004). Wang et al. (2018) found that NaCl (1.5 g NaCl/100) addition is helpful for the slurry to flow and becomes viscous post‐deposition, which was important for holding the shape of surimi‐gel. The result is supported by the work of Lin and Park (1996) who found that washing with higher amounts of NaCl solution reduced the loss of myofibrillar proteins that leads to an inferior gel‐forming ability (Shimizu et al., 1983). Washing facilitates the concentration of myofibrillar proteins, which constitute about 70% of the total proteins of fish meat and are the primary components of the formation of 3‐dimensional gel structure (Bakli et al., 2020). Hossain et al. (2004) also suggested that adding more salt during the washing process might cause a partial unfolding of proteins and increase the sensitivity to denaturation, causing a weaker gel matrix.

Furthermore, the presence of divalent cations such as Ca^2+^ and Mg^2+^ might strongly affect the strength, deformability, and interaction with protein molecules. Lertwittayanon et al. (2013) found that 0.45% NaCl washing solution containing CaCl_2_ or MgCl_2_ at various levels (0, 4, 8, 12, 16, and 20 mM) increases the gel‐forming ability of surimi produced from yellowtail barracuda. In the current study, the better textural quality of fish balls that came from mince washed with a divalent salt solution rather than a monovalent salt solution was also supported by the order of cation (Ca^2+^ >Mg^2+^ >Na^+^) for preferential interactions with proteins, where Na^+^ preferred to interact with water, and Ca^2+^ and Mg^2+^ preferred to bind with proteins via ionic interaction (Lertwittayanon et al., 2013). Washing is an important process for surimi production because it removes the undesirable water‐soluble proteins, blood, fat, and other nitrogenous components in fish mince and concentrates the myofibrillar proteins, improving the functional properties of the mince/surimi, (Bakli et al., 2020; Lin & Park, 1997). Thus, the use of appropriate salt solutions can be a way to improve the textural properties of surimi/mince‐based products such as fish balls. In addition, this study found that washing with different salt solutions had no significant effects on the color characteristics of the products (data not shown).

### 3.5 Proximate composition

The proximate composition of the fish, mince blend, and fish balls is presented in Table 6. The results showed that moisture content was similar in raw pangasius (69.75 ± 0.96%) and tuna (69.52 ± 0.58%) (*p* > .05). Based on the sensory and textural characteristics, the fish ball from the P_50_:T_50_ ratio was selected for proximate analysis. The moisture content of the unwashed mince blend and its resulted fish balls were 70.90 ± 0.96% and 70.49 ± 0.40%, respectively (*p* > .05). The moisture contained in the fish balls prepared from the mince blend washed with DW, 0.1% NaCl, and CaCl_2_ solution was 74.02 ± 0.33%, 74.43 ± 0.10%, and 75.13 ± 0.22%, respectively. Fish balls made from Indian mackerel (*Rastrelliger kanagurta*) contained moisture of 73.84% (Alkuraieef et al., 2020), and the moisture content of fish balls made from mackerel (*K. alvarezii*) varied between 75.66% and 77.89% (Asikin et al., 2020). This was significantly higher than the moisture contained in the fish balls from the unwashed mince (*p* < .05). The increase in moisture in the washed mince could be from the hydration of protein and the increase in the capacity to hold water through the formation of a 3‐dimensional network structure of heat‐induced gelation of myosin fish meat protein (Sun & Holley, 2011).

**TABLE 6:** Proximate composition of fish, mince blend, and fish balls

Sample	Proximate composition (%)			
	Moisture	Protein	Lipid	Ash
Pangasius	69.75 ± 0.96^d^	19.49 ± 0.71^bc^	8.84 ± 0.20^a^	1.71 ± 0.38^a^
Tuna	69.52 ± 0.58^d^	22.36 ± 0.97^a^	6.39 ± 0.19^b^	1.28 ± 0.21^a^
Unwashed mince blend	70.90 ± 0.96^c^	20.50 ± 0.25^b^	6.94 ± 0.41^b^	1.62 ± 0.33^a^
Fish balls from unwashed mince	70.49 ± 0.40 cd	18.95 ± 0.55^bc^	8.83 ± 0.87^a^	1.26 ± 0.47^a^
Fish balls from washed mince (DW)	74.02 ± 0.33^b^	17.74 ± 0.30^d^	6.71 ± 0.55^b^	1.26 ± 0.47^a^
Fish balls from washed mince (0.1% NaCl solution)	74.53 ± 0.10^ab^	17.95 ± 0.28^d^	5.88 ± 1.01^b^	1.57 ± 0.41^a^
Fish balls from washed mince (0.1% CaCl_2_ solution)	75.13 ± 0.22^a^	18.05 ± 0.21^d^	5.50 ± 0.91^b^	1.25 ± 0.30^a^

Note

Fish balls here were prepared with the selected mince blend ratio of P_50_:T_50_.

The protein content of pangasius and tuna was 19.49% and 22.36%, respectively. This result indicates that tuna contained significantly more protein than pangasius (*p* < .05). The protein content of the unwashed mince blend (P_50_:T_50_) and its resulted products were 20.50% and 18.95%, respectively (*p* > .05). Furthermore, the protein content of the resulted fish balls was found to be reduced significantly (17.74%–18.05%) when the mince blend was washed with DW, 0.1% NaCl, and CaCl_2_ solution (*p* < .05). However, there was comparatively lower protein content observed in mackerel fish ball (13.40%–15.23%) (Asikin et al., 2020) and Indian mackerel fish ball (12.94%) (Alkuraieef et al., 2020) than the current study. The fish species in this study contains higher protein in its raw state and that might result in higher protein in the mince‐blend fish ball. Between the washing solutions, the study did not observe any significant effects on the protein content (*p* > .05). On the other hand, the lipid contents of pangasius and tuna were 8.84% and 6.39%, respectively. The lipid content of the mince blend (P_50_:T_50_) was 6.94%. The lipid contents in the fish balls from the unwashed mince blend were 8.83%, which decreased significantly to 5.88%–6.71% when washed with different washing solutions (*p* < .05). Pangasius contained more lipids and less protein than tuna. The protein and lipid contents were higher in the unwashed mince than in the washed mince. Fish balls from washed mince prepared with substitution of rice bran and tapioca flour improved nutritional content (Affandi et al., 2019), and when incorporated with fish protein isolate, improved the protein and reduced the lipid contents (Ibrahim, 2015). In this study, an inverse relationship was found between the lipid content and moisture content. Higher moisture content in washed mince products (regardless of the solution) was co‐related with lower lipid content. Thammapat et al. (2010) reported an inverse proportion of protein, moisture, and ash content to lipids of *Pangasius bocourti*. Sarcoplasmic protein and lipids are a concern for mince or surimi‐based products due to their interference with the gel formation (Nowsad, Kanoh, et al., 2000). Washing the mince significantly reduces the sarcoplasmic protein, fat, blood, pigments, and enzymes from the mince blend (Sun & Holley, 2011). Hoque et al. (2007), Majumdar & Debbarma et al. (2013), and Yu (1994) also observed a significant loss of protein and lipids in washed mince during the preparation of value‐added fish products. On the other hand, similar ash content was observed in raw pangasius, raw tuna, and both washed and unwashed (with different solutions) mince blend (P_50_:T_50_) at 1.25%–1.71% (*p* > .05). The results indicate that the blend and the washing process had no significant effect on the minerals content of mince and its fish balls when using pangasius and tuna meat (*p* > .05). The different compositions of the respective two fish species might also contribute to the variation in the nutritional compositions of the mince blends. Blended fish mince taken from different species, sizes, sources, and seasonal variation may lead to the development of unique value‐added food products and thus could contribute to nutritional security.

## 4 CONCLUSION

An improved sensory, textural, and nutritional quality of fish balls were obtained from pangasius and tuna mince blends. Of the two species, tuna had higher total utilization than pangasius. Of the different mince blend ratios, the textural properties were better in fish balls from the mince blend of P_50_:T_50_ than that of other mince blend ratios. The two‐step heating process resulted in better textural quality than autoclaving. The tuna fish meat was responsible for the darker while pangasius mince blend resulted in whitish/brighter fish balls. Mince blend washed with different salt solutions (0.1% NaCl, KCl, CaCl_2_, and MgCl_2_) improved the textural quality of the fish balls compared to mince that was unwashed or washed with distilled water. The divalent cation solutions were more prominent to improve textural quality of fish ball than monovalent. Nutritionally, pangasius had higher lipid and lower protein content than tuna. Regardless of the species, protein and lipid contents were higher in unwashed mince than those in washed mince. Consequently, the mixed mince blend of (P_50_:T_50_) pangasius and tuna could favor higher nutritional and textural properties of fish balls. The study concluded that value‐added fish products based on different fish mince blends could ensure maximum resource utilization and nutritional security in Bangladesh or other countries.

## CONFLICT OF INTEREST

The authors declare no conflict of interest.

## ETHICAL APPROVAL

The study involved the human for sensory quality analysis of fish products which was conducted following the ethics approval from the Research and Training Center of Patuakhali Science and Technology University, Bangladesh.

## INFORMED CONSENT

Written informed consent was obtained from all study participants.

## Data Availability

Research data are not shared.
